# Gemcitabine promotes autophagy and lysosomal function through ERK- and TFEB-dependent mechanisms

**DOI:** 10.1038/s41420-023-01342-z

**Published:** 2023-02-06

**Authors:** Benoît Marchand, Marc-Antoine Poulin, Christine Lawson, Lee-Hwa Tai, Steve Jean, Marie-Josée Boucher

**Affiliations:** 1 Department of Medicine, Gastroenterology Division, Faculté de Médecine et des Sciences de la Santé, Université de Sherbrooke, Sherbrooke, Canada; 2 Department of Immunology and Cell Biology, Faculté de Médecine et des Sciences de la Santé, Université de Sherbrooke, Sherbrooke, Canada; 3 Member of the Centre de Recherche du CHUS and the Institut de recherche sur le cancer de l’Université de Sherbrooke, Sherbrooke, Canada

**Keywords:** Autophagy, Pancreatic cancer

## Abstract

Gemcitabine is a first-line treatment agent for pancreatic ductal adenocarcinoma (PDAC). Contributing to its cytotoxicity, this chemotherapeutic agent is primarily a DNA replication inhibitor that also induces DNA damage. However, its therapeutic effects are limited owing to chemoresistance. Evidence in the literature points to a role for autophagy in restricting the efficacy of gemcitabine. Autophagy is a catabolic process in which intracellular components are delivered to degradative organelles lysosomes. Interfering with this process sensitizes PDAC cells to gemcitabine. It is consequently inferred that autophagy and lysosomal function need to be tightly regulated to maintain homeostasis and provide resistance to environmental stress, such as those imposed by chemotherapeutic drugs. However, the mechanism(s) through which gemcitabine promotes autophagy remains elusive, and the impact of gemcitabine on lysosomal function remains largely unexplored. Therefore, we applied complementary approaches to define the mechanisms triggered by gemcitabine that support autophagy and lysosome function. We found that gemcitabine elicited ERK-dependent autophagy in PDAC cells, but did not stimulate ERK activity or autophagy in non-tumoral human pancreatic epithelial cells. Gemcitabine also promoted transcription factor EB (TFEB)-dependent lysosomal function in PDAC cells. Indeed, treating PDAC cells with gemcitabine caused expansion of the lysosomal network, as revealed by Lysosome associated membrane protein-1 (LAMP1) and LysoTracker staining. More specific approaches have shown that gemcitabine promotes the activity of cathepsin B (CTSB), a cysteine protease playing an active role in lysosomal degradation. We showed that lysosomal function induced by gemcitabine depends on TFEB, the master regulator of autophagy and lysosomal biogenesis. Interfering with TFEB function considerably limited the clonogenic growth of PDAC cells and hindered the capacity of TFEB-depleted PDAC cells to develop orthotopic tumors.

## Introduction

With the lowest 5-year survival rate among cancers, pancreatic ductal adenocarcinoma (PDAC) is a challenging cancer to treat. Although therapeutics have recently progressed, gemcitabine remains the mainstay of PDAC regimens [1, 2]. Nevertheless, the limited survival benefits of PDAC therapies underscore the urgent need to identify and test new therapeutic targets because predictions indicate that this cancer will become the second highest cause of cancer-related mortality by 2030 [1, 3, 4]. While waiting for the success of targeting common genetic alterations in PDAC, for example KRAS, other strategies are under evaluation such as interference with pancreatic cancer metabolic dependencies [5, 6].

Lysosomes play a central role in metabolic homeostasis by providing bioenergetic intermediates that fuel the metabolic pathways, thereby supporting the cellular energy demand [7–9]. Hydrolases, such as cathepsins, contained within these acidic organelles degrade cargo delivered *via* different routes. Among these, macroautophagy (herein referred to as autophagy) is responsible for escorting intracellular constituents engulfed in double-membrane vesicles into lysosomes [10]. Yang et al. discovered that autophagy levels are elevated in PDAC cells, which when inhibited pharmacologically or genetically, interfere with cell growth [11]. Thereafter, multiple studies using genetically engineered mouse models reinforced the notion that established pancreatic tumors depend on autophagy for growth [12–15]. In addition to autophagy, other cellular processes (such as macropinocytosis) also bring cargo to lysosomes. As the endpoint of different cargo routes, lysosomes thus serve as a final destination and are key in coordinating endocytic, phagocytic, and autophagic traffic [7–9]. Nonetheless, the importance of lysosome function in orchestrating degradative traffic to preserve homeostasis is just beginning to attract attention [7–9]. It is also most likely that lysosomes adapt to changes in traffic influx, particularly under pathological conditions and in response to environmental cues. Of interest, evidence supports the reliance of PDAC cells on the degradative lysosomal function as both autophagy and macropinocytosis are upregulated and contribute to tumor growth [6, 16, 17]. Nonetheless, a comprehensive picture of the mechanisms responsible for coordinating lysosomal function remains to be drawn.

Lysosome-related functions participate in chemoresistance [18] and interfering with autophagy sensitizes PDAC cells to gemcitabine [19–21]. However, these studies mainly focused on detecting lipidated LC3 (LC3-II) as an indicator of increased autophagy in response to gemcitabine, but the impact of gemcitabine on autophagic flux cannot be conclusively determined using only a single marker [22]. While the effects of gemcitabine on autophagy must be consolidated, its impact on lysosomal function remains unclear. More importantly, the mechanisms involved in these complementary, but distinct processes (autophagy and lysosomal function) must be identified to formulate strategies to overcome chemoresistance and augment the efficiency of cancer therapeutics for PDAC.

We found that gemcitabine did not sustainably impact the mammalian target of rapamycin complex 1 (mTORC1) signaling pathway, but triggered ERK-dependent autophagy in cancer cells and enhanced lysosomal function through TFEB-dependent mechanisms. The minimal clonogenic ability of TFEB-depleted cells incubated with gemcitabine indicated that this master regulator of autophagy and lysosomal function facilitates PDAC cells to cope more effectively with gemcitabine-induced stress. Furthermore, TFEB-depleted PDAC cells have limited capacity for orthotopic tumor growth. Overall, our findings support the rationale for targeting TFEB to counteract the protective and adaptive lysosome functions in PDAC and improve PDAC cell eradication, particularly when combined with gemcitabine as a first line treatment.

## Results

### Gemcitabine induces an autophagic response

**Fig. 1:**
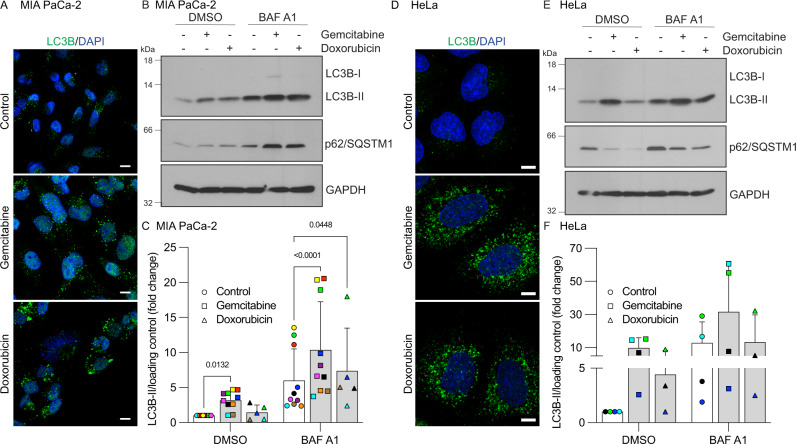
Gemcitabine promotes autophagy. **A** MIA PaCa-2 cells were incubated for 24 h with DMSO (control), gemcitabine (10 µM), or doxorubicin (0.5 µM) before autophagosome labeling (LC3B puncta). Nuclei were stained with DAPI. *Scale bars*, 10 µm. **B** MIA PaCa-2 cells were incubated for 48 h with vehicle (control; -), gemcitabine (10 µM), or doxorubicin (0.5 µM). Bafilomycin A1 (BAF A1; 50 nM) or vehicle (DMSO) was added 4 h prior to cell lysis. Total cell lysates were then analyzed by immunoblotting using LC3B, p62/SQSTM1, and GAPDH antibodies. **C** Graphical representation of LC3B-II levels quantified by immunoblotting (as in **B**) from MIA PaCa-2 biological replicates of 5‒10 color-coded independent experiments. The ratio of LC3B-II/loading control levels in control DMSO cells was set at 1. Data show color-coded independent experiments, means ± SD and were statistically analyzed using mixed model ANOVA with Tukey multiple comparison tests. **D** HeLa cells were incubated for 24 h with DMSO (control), gemcitabine (10 µM), or doxorubicin (0.5 µM) then autophagosomes were labeled (LC3B puncta). Nuclei were stained with DAPI. *Scale bars*, 10 µm. **E** HeLa cells were incubated for 48 h with vehicle (control; -), gemcitabine (10 µM), or doxorubicin (0.5 µM). Bafilomycin A1 (BAF A1; 50 nM) or vehicle (DMSO) was added 4 h prior to cell lysis. Total cell lysates were then analyzed by immunoblotting using LC3B, p62/SQSTM1, and GAPDH antibodies. **F** Graph of LC3B-II levels in HeLa cells quantified by immunoblotting (as in **E**) in biological replicates of 3-4 color-coded independent experiments. The ratio of LC3B-II/loading control levels in control DMSO cells was set at 1. Data show color-coded independent experiments, means ± SD.

To analyze the impact of gemcitabine on autophagy, the presence of autophagosomes was examined by detecting LC3B lipidation (LC3B-II). Immunofluorescence studies revealed that gemcitabine caused LC3B puncta (LC3B-II) to accumulate in the pancreatic cancer cell line MIA PaCa-2 (Fig.1A). Doxorubicin, another chemotherapeutic drug reported to impact autophagy [23, 24], also promoted the accumulation of LC3B puncta. Increased LC3B-II levels were also detected by immunoblotting in gemcitabine-treated MIA PaCa-2 cells (Fig. 1B, C). Autophagic flux was measured in cells incubated with the autophagy inhibitor bafilomycin A1 to distinguish the promotion of autophagosome formation from a blockade of lysosomal degradation, which could explain the accumulation of LC3B-II. Levels of LC3B-II were further increased in cells co-incubated with gemcitabine or doxorubicin compared with bafilomycin A1 alone (Fig. 1B, C). These findings indicated that gemcitabine and doxorubicin triggered autophagy.

p62/SQSTM1 is a protein adapter that directs selective cargo to autophagy-mediated degradation [22]. We monitored levels of p62/SQSTM1 as an indication of autophagic flux because it is degraded by autophagy. In untreated MIA PaCa-2 cells, p62/SQSTM1 levels were upregulated by bafilomycin A1, reflecting the basal levels of p62/SQSTM1 degraded by autophagy (Fig. 1B). Bafilomycin A1 also enhanced p62/SQSTM1 levels in cells incubated with gemcitabine and doxorubicin, again supportive of an autophagy response triggered by these agents.

To evaluate whether the impact of gemcitabine on autophagy was specific to pancreatic cancer MIA PaCa-2 cells, experiments were performed using HeLa cervical cancer cells. Increased LC3B puncta (Fig. 1D) and LC3B-II levels (Fig. 1E, F) were observed in HeLa cells incubated with gemcitabine. It is noteworthy that p62/SQSTM1 levels were reduced in the gemcitabine- and doxorubicin-treated HeLa cells, representing a typical result of autophagy-mediated p62/SQSTM1 degradation [22]. Inhibiting autophagy-mediated degradation by the addition of bafilomycin A1 promoted p62/SQSTM1 accumulation in control HeLa cells, and in those incubated with gemcitabine or doxorubicin (Fig. 1E). The reason why the regulation of p62/SQSTM1 slightly differed between MIA PaCa-2 and HeLa cells incubated with either gemcitabine or doxorubicin and without bafilomycin A1 remains elusive. However, the regulation of p62/SQSTM1 is complex, with transcriptional and post-translational regulatory mechanisms that are cell type- and context-specific [22, 25]. Nevertheless, an increase in p62/SQSTM1 levels upon autophagy inhibition, such as bafilomycin A1 addition, is suggestive of autophagy-mediated p62/SQSTM1 degradation and thus an indicator of autophagic flux [22, 26]. Overall, our results indicated that gemcitabine and doxorubicin trigger the autophagic response in various types of cancer cells.

### Gemcitabine promotes ERK activity

**Fig. 2:**
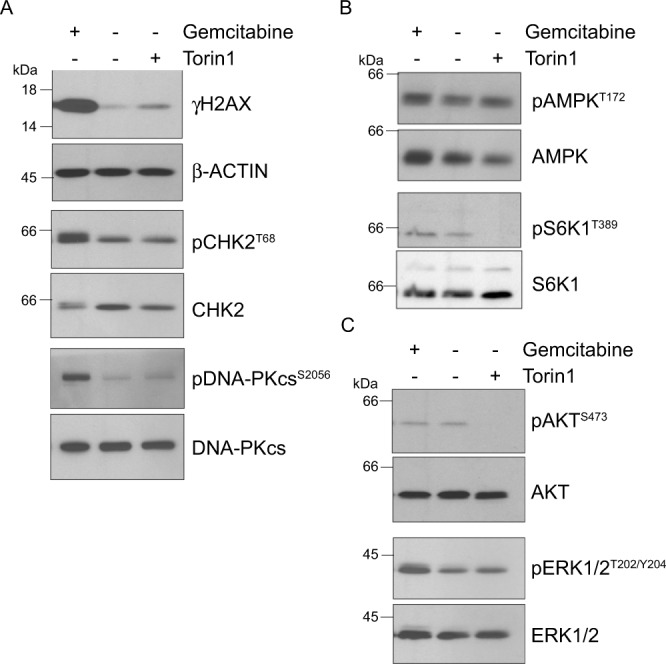
Gemcitabine promotes a DNA damage response and ERK activation. **A**‒**C** MIA PaCa-2 cells were incubated for 24 h with vehicle (−), gemcitabine (10 µM), or Torin1 (250 nM). Total cell lysates were analyzed by immunoblotting using the indicated antibodies.

Gemcitabine induces DNA damage, which was confirmed herein as elevated levels of γH2AX (Fig. 2A). Furthermore, gemcitabine increased the phosphorylation levels of CHK2 and DNA-PKcs that are both involved in the DNA damage response. Conversely, Torin1, an mTOR inhibitor that serves as a control for autophagy induction, did not significantly affect γH2AX, or CHK2 and DNA-PKcs phosphorylation levels (Fig. 2A). Of particular interest, accumulating evidence suggests that autophagy is elicited upon DNA damage [27].

Although AMPK and mTORC1 signaling are well-known regulators of autophagy, no clear modulation in their phosphorylation levels, as a readout for their activities, was detected upon gemcitabine treatment. Notably, Torin1 efficiently hampered S6K1 phosphorylation, a downstream target of mTORC1 (Fig. 2B). These results indicated that gemcitabine most likely exploits signaling pathways other than AMPK or mTORC1 to modulate autophagy.

**Fig. 3:**
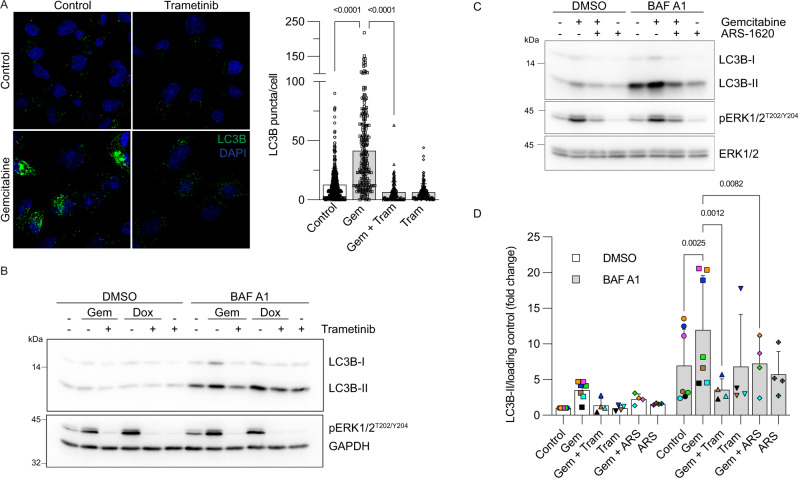
Blockade of MEK–ERK signaling prevents gemcitabine-induced autophagy. **A** MIA PaCa-2 cells were incubated for 24 h with vehicle (control), gemcitabine (Gem; 10 µM), and/or trametinib (Tram; 100 nM) before LC3B labeling (as in Fig. 1A). Numbers of LC3B puncta per cell were calculated using CellProfiler software. Scatter dot plot shows data as means ± SD; *n* = 114–419 cells from 3–6 independent experiments. Data were statistically analyzed using one-way ANOVA with Tukey *post hoc* tests. **B** MIA PaCa-2 cells were incubated for 48 h with vehicle (−), gemcitabine (Gem; 10 µM), and doxorubicin (Dox; 0.5 µM) with or without trametinib (100 nM). Bafilomycin A1 (BAF A1; 50 nM) or vehicle (DMSO) was added 4 h prior to cell lysis. Total cell lysates were then immunoblotted using indicated antibodies. **C** MIA PaCa-2 cells were incubated for 48 h with vehicle (−) or gemcitabine (10 µM), with or without ARS-1620 (10 µM). Bafilomycin A1 (BAF A1; 50 nM) or vehicle (DMSO) was added 4 h prior to cell lysis. Total cell lysates were immunoblotted with indicated antibodies. **D** Graphical representation of LC3B-II levels quantified by immunoblotting (as in Fig. 3B, C) from MIA PaCa-2 biological replicates of 4‒7 color-coded independent experiments. The ratio of LC3B-II/loading control levels in control DMSO cells was set at 1. Data show color-coded independent experiments, means ± SD and were statistically analyzed using mixed model ANOVA with Tukey multiple comparison tests.

We assessed activities of the PI3K-AKT and MEK-ERK signaling pathways because both are thought to influence PDAC cell metabolic dependence, including autophagy [28–30]. Unlike Torin1, gemcitabine did not impact AKT phosphorylation. However, gemcitabine consistently upregulated ERK1/2 phosphorylation (Figs. 2C, 3B, C). Notably, doxorubicin also stimulated ERK1/2 activity (Fig. 3B).

### Blocking RAS-RAF-MEK-ERK signaling prevents gemcitabine-induced autophagy

To evaluate whether gemcitabine-induced ERK1/2 activity contributed to the autophagic response, cells were incubated with the specific MEK1/2 inhibitor trametinib [31]. Trametinib hindered basal, gemcitabine- or doxorubicin-induced ERK1/2 phosphorylation (Fig. 3B). Trametinib abrogated the LC3B puncta accumulation induced by gemcitabine (Fig. 3A) and inhibited the autophagy flux induced by gemcitabine and doxorubicin, as evidenced by reduced LC3B-II levels in cells incubated with bafilomycin A1 (Fig. 3B, D).

**Fig. 4:**
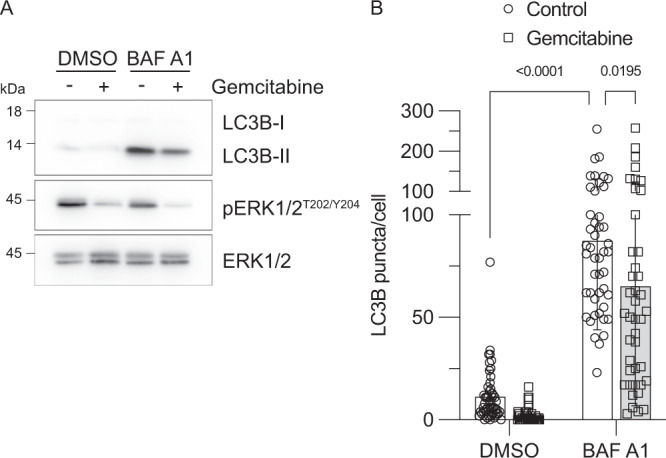
Gemcitabine does not increase ERK activities or autophagy flux in non-tumoral pancreatic epithelial cells. **A** HPDE cells were incubated 48 h with vehicle (−) or gemcitabine (5 µM). Bafilomycin A1 (BAF A1; 50 nM) or vehicle (DMSO) was added 4 h prior to cell lysis. Total cell lysates were then immunoblotted with indicated antibodies. **B** HPDE cells were incubated for 24 h with vehicle (Control) or gemcitabine (5 µM). Bafilomycin A1 (BAF A1; 50 nM) or vehicle (DMSO) was added 4 h prior to LC3B labeling. The numbers of labeled LC3B puncta per cell were calculated using CellProfiler software. Scatter dot plot shows means ± SD; *n* = 42–52 cells from two independent experiments. Data were statistically analyzed using two-way ANOVA with Tukey *post hoc* tests.

The MIA PaCa-2 cell line harbors a KRAS^G12C^ mutation. We therefore assessed the effects of the KRAS^G12C^ inhibitor ARS-1620 to clarify the role of the MEK–ERK pathway downstream of RAS signaling in gemcitabine-induced autophagy. ARS-1620 limited gemcitabine-induced ERK phosphorylation and autophagy flux, thus reinforcing the contribution of the RAS–ERK pathway in gemcitabine-induced autophagy (Fig. 3C, D). Gemcitabine notably did not increase ERK1/2 phosphorylation in non-tumoral pancreatic epithelial (HPDE) cells (Fig. 4A). Measurement of autophagic flux with bafilomycin A1 revealed lower levels of LC3B-II and LC3B puncta in HPDE cells incubated with gemcitabine (Fig. 4A, B). These results suggest that the mechanisms leading to ERK activation and promotion of autophagy upon gemcitabine treatment in pancreatic cancer cells are not operational in HPDE cells.

### Gemcitabine promotes lysosomal function

**Fig. 5:**
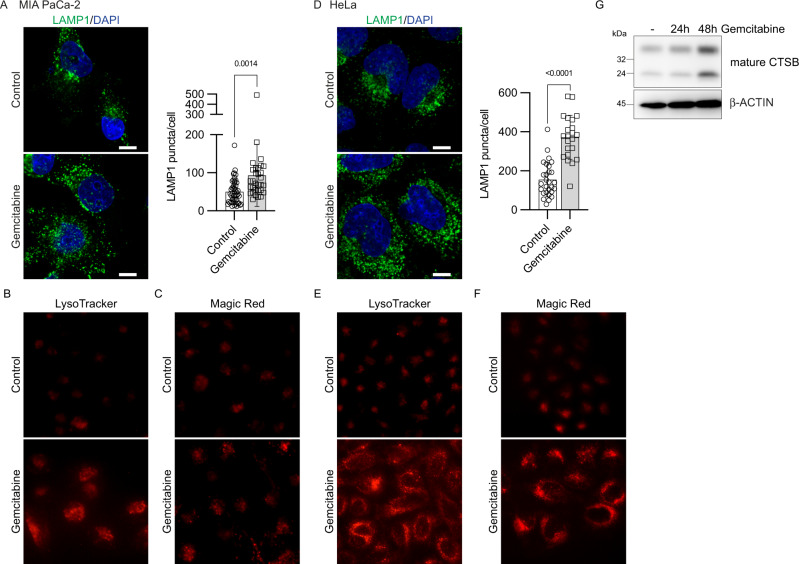
Gemcitabine promotes lysosomal function. **A** MIA PaCa-2 cells were incubated for 24 h with vehicle (Control) or gemcitabine (10 µM) before lysosome labeling using anti-LAMP1 antibody. Nuclei were stained with DAPI. *Scale bars*, 10 µm. Graph shows numbers of LAMP1 puncta per cell calculated using CellProfiler software. Scatter dot plot shows data as means ± SD; *n* = 31‒48 cells from three independent experiments. Data were statistically analyzed using unpaired t-tests. **B** Representative images of live MIA PaCa-2 cells incubated with vehicle (Control) or gemcitabine (10 µM) for 24 h then stained with LysoTracker. **C** Representative images of live MIA PaCa-2 cells incubated with vehicle (Control) or gemcitabine (10 µM) for 24 h then stained with Magic Red. **D** HeLa cells were incubated for 24 h with vehicle (Control) or gemcitabine (10 µM) before lysosome labeling using anti-LAMP1 antibody. Nuclei were stained with DAPI. *Scale bars*, 10 µm. Graph shows numbers of LAMP1 puncta per cell calculated using CellProfiler software. Scatter dot plot shows data as means ± SD; *n* = 22–28 cells from three independent experiments. Data were statistically analyzed using unpaired *t*-tests. **E** Representative images of live HeLa cells incubated with vehicle (Control) or gemcitabine (10 µM) for 24 h then stained with Lysotracker. **F** Representative images of live HeLa cells incubated with vehicle (Control) or gemcitabine (10 µM) for 24 h then stained with Magic Red. **G** MIA PaCa-2 cells were incubated with vehicle (−) or gemcitabine (10 µM) for the indicated periods. Total cell lysates were analyzed by immunoblotted using anti-CTSB and β-ACTIN antibodies. Levels of pro-CTSB are barely detectable compared with mature CTSB.

In accordance with the enhanced autophagy capacity, an expanded lysosomal network, as measured by LAMP1 staining, was detected in MIA PaCa-2 and HeLa cells incubated with gemcitabine (Fig. 5A, D). Using the LysoTracker dye, labeling of acidic organelles i.e. lysosomes was amplified in cells incubated with gemcitabine (Fig. 5B and E). Similarly, using the Magic Red substrate, increased lysosomal CTSB activity was detected in cells incubated with gemcitabine (Fig. 5C, F). Furthermore, immunoblots revealed increased levels of mature CTSB in cells incubated with gemcitabine (Fig. 5G). These results indicated that gemcitabine expands the network of functional lysosomes.

### Gemcitabine promotes TFEB nuclear localization

**Fig. 6:**
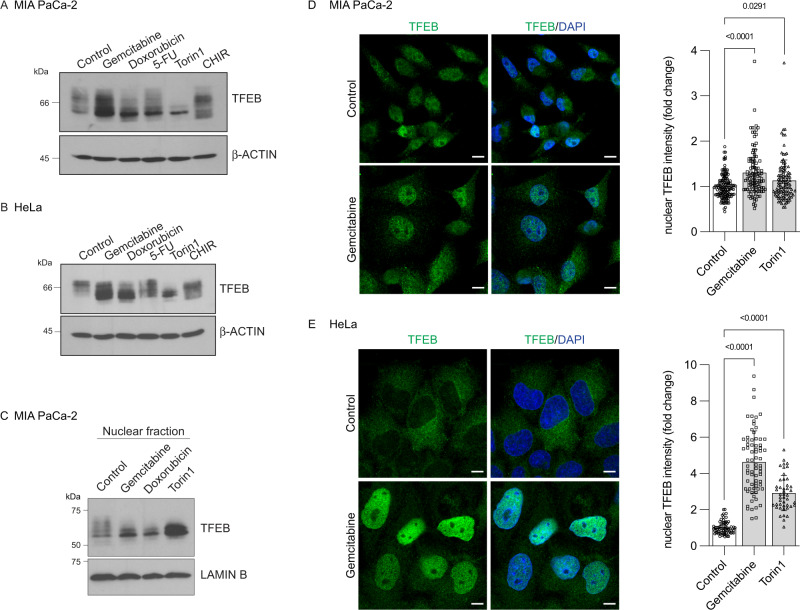
Gemcitabine promotes TFEB nuclear localization. **A**, **B** MIA PaCa-2 (**A**) and HeLa (**B**) cells were incubated for 24 h with vehicle (control), gemcitabine (10 µM), doxorubicin (0.5 µM), 5-FU (100 µM), Torin1 (250 nM), or CHIR99021 (CHIR; 5 µM). Total cell lysates were immunoblotted using indicated antibodies. **C** MIA PaCa-2 cells were incubated for 24 h with vehicle (control), gemcitabine (10 µM), doxorubicin (0.5 µM), or Torin1 (250 nM). Nuclear fractions were prepared and analyzed by immunoblotting using anti-TFEB and anti-LAMIN B antibodies. **D** MIA PaCa-2 cells were incubated for 24 h with vehicle (Control), gemcitabine (10 µM) or Torin1 (250 nM) before analysis of TFEB by immunofluorescence. Nuclei were stained with DAPI. *Scale bars*, 10 µM. Representative images are shown for the control and gemcitabine-treated cells. Graph shows the nuclear median integrated intensity of TFEB immunofluorescence emitted by 105–137 cells from four independent experiments. Data were statistically analyzed using one-way ANOVA with Dunnett *post hoc* tests. **E** HeLa cells were incubated for 24 h with vehicle (Control), gemcitabine (10 µM) or Torin1 (250 nM) before analysis of TFEB by immunofluorescence. Nuclei were stained with DAPI. *Scale bars*, 10 µM. Representative images are shown for the control and gemcitabine-treated cells. Graph shows the nuclear TFEB median integrated intensity of 45–72 cells from four independent experiments. Data were statistically analyzed using one-way ANOVA with Dunnett *post hoc* tests.

The microphthalmia/transcription factor E (MiT/TFE) family of transcription factors, particularly TFEB, is considered a master regulator of autophagy and lysosomal function [7–9]. We therefore investigated their contribution to gemcitabine-induced lysosomal function. Although several regulatory mechanisms remain to be elucidated, the typical representation of TFEB regulation consists of the phosphorylated transcription factor being in the cytoplasm. In contrast, dephosphorylation events, translated into an acceleration in electrophoretic mobility, allow TFEB to accumulate within the nucleus [32]. Figure 6A shows that, in MIA PaCa-2 cells, gemcitabine, doxorubicin and 5-FU altered the electrophoretic mobility of TFEB with enriched lower molecular weight forms. The migration pattern of TFEB was distinct when cells were incubated with Torin1, the inhibition of the mTOR pathway being established to promote the accumulation of dephosphorylated TFEB [32]. The electrophoretic mobility of TFEB was also distinct when GSK3 was inhibited by CHIR99021 as we previously found [33]. These results implied that various stressors differently impact TFEB electrophoretic mobility. In HeLa cells, Gemcitabine, doxorubicin and 5-FU similarly modulated TFEB phosphorylation determined as altered TFEB electrophoretic mobility (Fig. 6B).

To correlate the acceleration in electrophoretic mobility with TFEB nuclear enrichment, subcellular fractionation and immunofluorescence studies were performed. Gemcitabine and doxorubicin both increased TFEB levels in the nuclear fraction compared with control cells (Fig. 6C). Furthermore, gemcitabine enriched nuclear TFEB staining in MIA PaCa-2 compared with control cells (Fig. 6D). This was despite pancreatic cancer cells already displaying some extent of TFEB nuclear localization under basal conditions as previously reported [33–36]. The effects of gemcitabine on TFEB shuttling and nuclear accumulation were better appreciated in HeLa cells, in which TFEB localized in the cytoplasm under basal (control) conditions (Fig. 6E). Overall, our results indicated that gemcitabine promotes TFEB nuclear enrichment.

### TFEB limits gemcitabine-induced lysosomal function and confers a growth advantage to pancreatic cancer cells

**Fig. 7:**
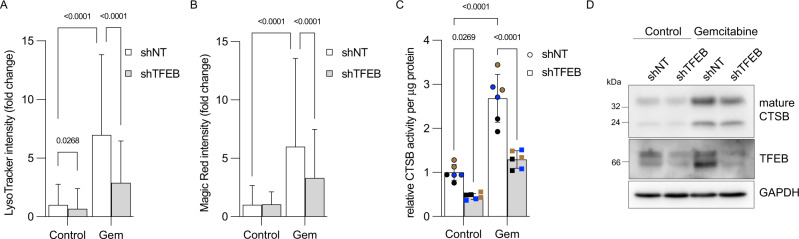
Interfering with TFEB expression impairs gemcitabine-induced lysosomal function. **A** Quantification of MIA^shNT^ and MIA^shTFEB^ cells incubated for 24 h with vehicle (Control) or gemcitabine (Gem; 10 µM) and stained with LysoTracker. Data are shown as means ± SD of *n* = 471–2196 cells from three independent experiments and were statistically analyzed using two-way ANOVA with Tukey *post hoc* tests. **B** Quantification of MIA^shNT^ and MIA^shTFEB^ cells incubated for 24 h with vehicle (Control) or gemcitabine (Gem; 10 µM) and stained with Magic Red. Data are shown as means ± SD of *n* = 1220–3156 cells from three independent experiments and were statistically analyzed using two-way ANOVA with Tukey *post hoc* tests. **C** Activity of CTSB in MIA^shNT^ and MIA^shTFEB^ cells incubated with vehicle (Control) or gemcitabine (Gem; 10 µM) for 24 h (*N* = 6 from three color-coded independent experiments). Data show color-coded independent experiments, means ± SD and were statistically analyzed using two-way ANOVA with Tukey *post hoc* tests. **D** MIA^shNT^ and MIA^shTFEB^ cells were incubated for 48 h with vehicle (Control) or gemcitabine (10 µM). Total cell lysates were analyzed by immunoblotting using anti-TFEB, CTSB, and GAPDH antibodies.

To test whether TFEB contributes to gemcitabine-induced lysosomal function, MIA PaCa-2 cells stably depleted of TFEB through an shRNA strategy (MIA^shTFEB^) were used. LysoTracker staining revealed fewer acidic lysosomes in MIA^shTFEB^ than MIA^shNT^ cells incubated with gemcitabine (Fig. 7A). Furthermore, the results of various assays supported reduced lysosomal CTSB activity in cells depleted of TFEB. Indeed, although untreated MIA^shTFEB^ cells did not display a significant decrease in Magic Red intensity when compared to the corresponding intensity measured in the control population (MIA^shNT^), the gemcitabine-induced Magic Red intensity was significantly abrogated in MIA^shTFEB^ (Fig. 7B). Furthermore, fluorometric assays revealed reduced CTSB activity in gemcitabine-treated MIA^shTFEB^ cells compared with the CTSB activity observed in gemcitabine-treated MIA^shNT^ cells (Fig. 7C). Accordingly, immunoblots revealed lower levels of mature CTSB in MIA^shTFEB^ cells (Fig. 7D). Taken together, these results indicated that gemcitabine induces TFEB-dependent lysosomal function.

**Fig. 8:**
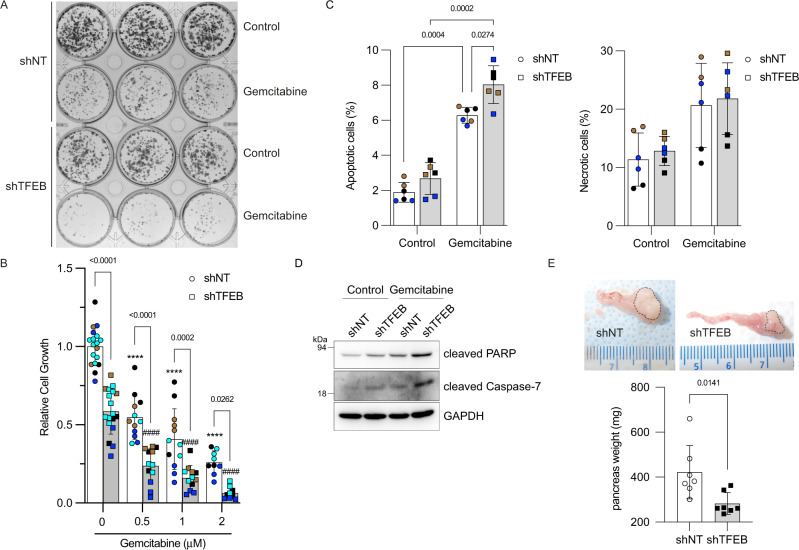
TFEB confers a growth advantage to pancreatic cancer cells. **A** Representative clonogenic assays of MIA^shNT^ and MIA^shTFEB^ cells pulse-treated 6 h with vehicle (Control) or gemcitabine (2 µM). **B** Quantification of clonogenic assays for MIA^shNT^ and MIA^shTFEB^ cells pulse-treated 6 h with the indicated concentrations of gemcitabine. Scatter dot plot shows means ± SD; *N* = 9–18 biological replicates from three to four color-coded independent experiments. Data were statistically analyzed using two-way ANOVA with Tukey *post hoc* tests as indicated and ****p < 0.0001 when compared with control MIA^shNT^, ####*p* < 0.0001 when compared with control MIA^shTFEB^. **C** MIA^shNT^ and MIA^shTFEB^ cells were incubated for 24 h with vehicle (Control) or 10 µM gemcitabine and cell death was analyzed by the Annexin V-PE detection kit and quantified by flow cytometry as described in materials and methods. Scatter dot plot shows data as means ± SD for apoptotic (early and late) and necrotic cells; *N* = 6 from three color-coded independent experiments. Data were statistically analyzed using two-way ANOVA with Tukey *post hoc* tests. **D** MIA^shNT^ and MIA^shTFEB^ cells were incubated for 24 h with vehicle (Control) or gemcitabine (10 µM). Total cell lysates were analyzed by immunoblotting using the indicated antibodies. **E** Representative images of pancreas (dashed lines indicate tumor) from mice orthotopically injected with MIA^shNT^ and MIA^shTFEB^ cells. Graph shows the average weight of the pancreas from these mice. Scatter dot plot shows means ± SD (*N* = 7 mice/group). Data were statistically analyzed using unpaired t-tests.

To assess whether interference with TFEB function enhances the response to gemcitabine, we performed clonogenic assays. Similar to PANC1^shTFEB^ cells that have reduced anchorage-independent growth ability compared with the corresponding growth ability in their control counterpart [33], MIA^shTFEB^ cells were nearly half capable of forming colonies compared with MIA^shNT^ cells (Fig. 8A, B). Upon gemcitabine treatment, cell growth was significantly impaired in both MIA^shNT^ and MIA^shTFEB^ cells, with the latter exhibiting limited growth ability following treatment with 2 μM gemcitabine (Fig. 8B). Therefore, gemcitabine together with disrupted TFEB function considerably hindered PDAC cell growth.

We used flow cytometry to determine whether the limited ability of TFEB-depleted cells to grow under gemcitabine was a consequence of increased cell death. We observed a higher percentage of apoptotic cells in gemcitabine-treated MIA^shTFEB^ cells when compared with gemcitabine-treated MIA^shNT^ cells, whereas no significant effect on the percentage of necrotic cells was detected (Fig. 8C). Immunoblotting revealed higher levels of the apoptotic markers cleaved PARP and caspase-7 in MIA^shTFEB^, than in MIA^shNT^ cells incubated with gemcitabine (Fig. 8D). These results overall suggested that interference with TFEB limits PDAC cell growth and promotes gemcitabine-induced apoptosis.

We assessed whether MIA^shTFEB^ cells could develop orthotopic tumors to further support the notion that TFEB plays a significant role in PDAC growth. The impact of TFEB knockdown and its family members TFE3 and MITF on PDAC tumor growth has been evaluated in subcutaneous xenografts [35, 37], but not in orthotopic models, that better recapitulate human disease. We found that tumors were significantly smaller in MIA^shTFEB^, than MIA^shNT^ cells, indicating that TFEB contributes to PDAC tumor growth (Fig. 8E).

## Discussion

Supported by the direct measurement of autophagy flux, our study consolidates the notion that gemcitabine induces an autophagic response in PDAC cells [19–21, 38]. One novelty of our analysis is the demonstration that this response includes the ERK signaling pathway. In accordance with ERK activation participating in gemcitabine-induced autophagy, non-tumoral pancreatic epithelial HPDE cells did not respond to gemcitabine with increased ERK activity or autophagy. Further studies are required to reveal the mechanisms leading to gemcitabine-induced ERK activation, as this may involve context-dependent direct and/or indirect mechanisms.

Our results align well with initial correlations between oncogenic RAS signaling and increased autophagy [11, 39–41]. Consistent with a supporting role for the ERK pathway in autophagy, HPDE cells stably expressing an oncogenic KRAS had elevated ERK activity and basal autophagy (data not shown), results that are similar to those of Maertin et al. [42]. In contrast, an autophagic response has been identified in PDAC cells incubated with an ERK inhibitor, implying that restricting ERK promotes autophagy [29, 30], which is contradictory to our findings. However, we assessed the impact of ERK inhibition combined with gemcitabine, whereas these studies did not. Given that gemcitabine stimulates ERK1/2 and the degree of ERK activity dictates cell responses to the same stimuli [43–45], the autophagy response might depend on ERK activity levels. Our results were still somewhat consistent with theirs in that stress triggers an autophagic response in PDAC cells. Gemcitabine, as a DNA replication inhibitor and inducer of DNA damage, most likely acted as a stressor in the present study. Conversely, Bryant et al. proposed that the significant and persistent ERK inhibition triggered by the MEK inhibitor leads to metabolic stress as a result of reduced glycolysis, which causes cells to become more reliant on autophagy for providing metabolic intermediates [29]. The same group recently found that, opposingly, increased ERK1/2 activity correlates with autophagy induction in PDAC cells incubated with a CHK1 inhibitor; [46] this emphasized a complex relationship between ERK activity and autophagy regulation. The kinetics of the autophagy response triggered by gemcitabine in the present study provided further support for the notion that PDAC cells adapt to cellular stressors by promoting autophagy. Incubating cells for 24 h with gemcitabine obviously increased LC3B and LAMP1 puncta, and the intensity of LysoTracker and Magic Red. Still, the direct measurement of autophagy flux with bafilomycin A1 addition led to more compelling results after 48 h incubation with gemcitabine, suggesting that the autophagy (and lysosomal response) and potentially ERK activation may be the results of the gemcitabine-imposed stress rather than a direct effect.

Our findings draw particular attention to the promotion of lysosomal function by gemcitabine. Recent electron microscopy findings have shown that gemcitabine increases the intracellular abundance of lysosomes [47]. In agreement with this, LysoTracker staining revealed that gemcitabine increased the numbers of acidic lysosomes in cells in the present study. More specific approaches for testing lysosomal function showed that gemcitabine stimulated lysosomal CTSB activity, and that this effect was significantly attenuated in cells with reduced TFEB expression. These findings attested to the involvement of TFEB in the regulation of lysosomal function, particularly under stress. Depleting TFEB critically limited gemcitabine-induced, but not basal lysosomal function. One explanation could be that gemcitabine impacts TFEB post-translational modifications and promotes its nuclear localization. Therefore, phosphorylated TFEB might play a limited role under basal conditions. In contrast, TFEB might contribute more to lysosomal function under stimulation with stressors such as gemcitabine that modify TFEB phosphorylation profile and promotes its nuclear enrichment. In support of a critical role of TFEB under stress, TFEB depletion reduces anchorage-independent [33], clonogenic, and tumor (present study) growth despite the absence of a significant effect when cultured under normal 2D conditions. Furthermore, the clonogenic growth of TFEB-depleted cells was minimal under additional stress imposed by gemcitabine. Although gemcitabine combined with TFEB targeting requires evaluation in vivo, our data underscored the promise of TFEB as a relevant therapeutic target, especially in the context of PDAC. Indeed, the MiT/TFE family of transcription factors are candidate contributors to PDAC pathophysiology [35, 48]. Notably, TFEB and TFE3 expression levels are increased in pancreatic cancer, compared with normal pancreatic tissues [34, 35]. Moreover, aberrant nuclear localization of MiT/TFE transcription factors is a common feature of pancreatic cancer cells suggesting that they assume critical function in PDAC [33–36].

Our results emphasized the role of TFEB in the regulation of lysosomal function. Interestingly, PDAC tumors exploit autophagy and macropinocytosis to scavenge nutrients [6, 49], both of which increase the cargo load managed by lysosomes. Evidence indicates that PDAC cells are equipped to face this cargo overload by adapting and modifying their lysosome membrane composition [50]. However, the mechanisms leading to these changes remain unknown. It will be interesting to assess the perspective that TFEB participates in transcriptional reprogramming that results in improved lysosomes, which enable PDAC cells to withstand increased levels of cellular stress such as the one imposed by gemcitabine.

Overall, our findings that MEK inhibition prevented gemcitabine-induced autophagy, not only facilitated identification of a mechanism through which gemcitabine promotes autophagy but also emphasized the complex relationship between the ERK signaling pathway and autophagy, especially in PDAC cells. Further studies are warranted to decipher the mechanisms through which ERK signaling fine-tunes autophagy. From a therapeutic perspective, it will be challenging to reach a precise level of ERK activity in cancer cells in vivo, particularly in terms of combination therapy. This precise degree of ERK activity will need to correlate with autophagy inhibition. Furthermore, RAS- and MEK-ERK-targeted therapies have been the focus of PDAC therapeutic development over the long term [51], but have not yet demonstrated the anticipated benefit. Considering that resistance mechanisms develop under RAS or MEK inhibitors even in PDAC [52], refining RAS or MEK targeting might be lengthy and laborious. We believe that a better understanding of TFEB regulation and function in PDAC pathophysiology is vital. It might offer an attractive PDAC target at the crossroads of the essential autophagy and macropinocytosis processes that converge on lysosomes and play recognized roles in PDAC pathophysiology. The potential therapeutic benefit of disrupting autophagy and lysosome function has been trialed in patients with metastatic PDAC. However, adding hydroxychloroquine to the gemcitabine and nanoparticle albumin-bound-paclitaxel regimen did not improve 1-year overall survival rates [53]. Given that hydroxychloroquine promotes TFEB nuclear localization [54] like gemcitabine, and that TFEB confers a growth advantage on PDAC cells, directly hijacking TFEB as a strategy to hamper lysosome function might be more effective if combined with current PDAC regimens. Whether restricting TFEB function reverses the beneficial changes on the lysosome membrane that help cells to resist stress [50] would be important to know. Consequently, PDAC cells would become less resistant to a hostile microenvironment, resulting in limited tumor growth and increased vulnerability to stress that requires lysosome function for recovery. Noteworthy, autophagy/lysosome function plays an important role in recovery from replication stress [55] with the latter representing the primary mechanism through which gemcitabine interferes with cell growth. As transcription factors can now being targeted and might represent a strategy to limit the development of resistance [56], TFEB deserves considerable attention. Overall, the present findings provide robust evidence that TFEB governs lysosomal function in PDAC cells, particularly under stress. We showed that TFEB targeting remarkably hampers PDAC cell growth when combined with the first-line therapeutic agent gemcitabine.

## Materials and methods

### Cell culture and drugs

HeLa and MIA PaCa-2 cells were obtained from ATCC and cultured in DMEM supplemented with 10% fetal bovine serum (Wisent Bioproducts, St-Bruno, QC, Canada), 10 mM HEPES (Wisent Bioproducts), and 2 mM GlutaMAX^TM^ (Thermo Fisher Scientific Inc., Mississauga, ON, Canada) at 37 °C under a humidified 5% CO_2_ atmosphere. The generation of MIA PaCa-2 cells stably expressing a non-targeting shRNA (shNT) or shRNA targeting TFEB (shTFEB) was identical to the method we described for PANC1 [33]. Non-tumoral human pancreatic duct epithelial (HPDE) cells were kindly provided by Ming-Sound Tsao (University of Toronto, ON, Canada) and cultured in keratinocyte Serum-Free Growth Medium (SFM) (Thermo Fisher Scientific Inc.) as described [33, 57, 58]. Cells were incubated with gemcitabine (LC Laboratories, Woburn, MA, USA), doxorubicin (LC Laboratories), or 5-fluorouracil (5-FU; Sigma-Aldrich Canada Corp., Oakville, ON, Canada) for the indicated periods. The following inhibitors were purchased from the listed suppliers: the mTOR inhibitor Torin1 and GSK3 inhibitor CHIR99021 (Selleck Chemicals LLC., Houston, TX, USA), MEK inhibitor trametinib, and the KRAS^G12C^ inhibitor ARS-1620 (MedChemExpress, Monmouth Junction, NJ, USA), and Bafilomycin A1 (LC Laboratories).

### Immunoblotting

The cells were rinsed with ice-cold PBS and lysed in RIPA buffer (1% NP40, 50 mM Tris, 150 mM NaCl, 0.5% sodium deoxycholate, 0.1% SDS, 5 mM EDTA, 10 mM NaF, 40 mM β-glycerophosphate, 5% glycerol, 0.5 µg/mL aprotinin, 0.5 µg/mL leupeptin, 0.7 µg/mL pepstatin, 1 mM PMSF, 200 µM orthovanadate). Total cell lysates were sonicated, and cellular debris was cleared by centrifugation (13,000 g, 10 min, 4 °C). Protein concentrations were quantified using the bicinchoninic acid (BCA) reagent procedure (Thermo Fisher Scientific Inc.), with bovine serum albumin as the standard. Equal amounts of proteins were separated by SDS-PAGE, transferred onto Amersham PVDF membranes (Sigma-Aldrich Canada Corp.), and immunoblotted as described [33, 59].

Nuclear fractions were prepared using commercial kits (#9038; New England Biolabs Ltd, Whitby, ON, Canada) as described by the manufacturer.

The following antibodies were purchased from Cell Signaling Technology (New England Biolabs Ltd): Microtubule-associated proteins 1 A/1B light chain 3B (LC3B; #3868), Sequestome 1 (p62/SQSTM1; #8025), Glyceraldehyde 3-phosphate dehydrogenase (GAPDH; #2118), phosphorylated Checkpoint kinase 2 (pCHK2^T68^; #2197), AMP-activated protein kinase (AMPK; #5832), phosphorylated AMPK (pAMPK^T172^; #2535), ribosomal protein S6 kinase beta-1 (S6K1; #2708), phosphorylated S6K1 (pS6K1^T389^; #9234), cellular homolog of murine thymoma virus akt8 oncogene (AKT; #9272), phosphorylated AKT (pAKT^S473^; #9271), phosphorylated extracellular signal-regulated kinase (pERK1/2^T202/Y204^; #9101), TFEB (#4240), CTSB (#31718), cleaved caspase-7 (#9491) and cleaved poly-ADP ribose polymerase (cleaved PARP; #5625). The following antibodies were purchased from the respective suppliers: LAMIN B (sc-6217), Lysosome associated membrane protein-1 (LAMP1; sc-20011), gamma H2A histone family member X (γH2AX; sc-101696), CHK2 (sc-5278), DNA-dependent protein kinase, catalytic subunit (DNA-PKcs; sc-390849), and ERK (sc-93) from Santa Cruz Biotechnology (Dallas, TX, USA); anti-pDNA-PKcs^S2056^ (#18192) from Abcam; anti-β-ACTIN (MAB1501R) from MilliporeSigma (Oakville, ON, Canada); horseradish peroxidase (HRP)-conjugated anti-mouse (115-035-003) and anti-rabbit IgG (111-035-003) from Jackson ImmunoResearch Laboratories Inc. (West Grove, PA, USA).

### Immunofluorescence and confocal microscopy

Cells were seeded on 8-well chambered culture slides (Corning Inc., Corning, NY, USA) for 24 h before being treated as indicated. The cells were then washed in PBS and fixed with 2% paraformaldehyde for 15 min at room temperature. The cells were permeabilized with either 0.3% Triton or 100% methanol (-20 °C) for 10 min depending on the target primary antibody. Then, non-specific antigen binding was blocked by incubation for 1 h at room temperature with 5% bovine serum albumin in PBS containing 0.1% TWEEN (blocking solution). The cells were incubated with primary antibodies diluted in blocking solution overnight at 4 °C, washed in PBS containing 0.1% TWEEN then incubated at room temperature for 1 h with the fluorescence-tagged secondary antibodies goat anti-mouse or anti-rabbit DyLight 488 or 549 (Vector Laboratories Inc., Newark, CA, USA) diluted in blocking solution. Thereafter, the cells were washed and stained with DAPI, then mounted on slides using Fluoromount G (Thermo Fisher Scientific Inc.). Images were acquired with constant settings using ZEN 2.5 (blue edition) software and a ZEISS LSM 880 confocal laser scanning microscope equipped with a Plan-Apochromat 40×/1.4 oil DIC M27 objective at 1.5‒3× zoom (All from Carl Zeiss, AG., Oberkochen, Germany). Z-sections (*N* = 3‒5) were acquired at intervals of 0.33–0.49 μm, and the results of maximum intensity projections are shown.

Images were analyzed using CellProfiler (v. 3.1.9), and a pipeline was created to quantify punctate LC3B or LAMP1. Briefly, the images were converted to grayscale and prepared for object identification. Nuclei were detected using a set of modules applying a global threshold to the grayscale-DAPI images, proceed with majority and bridge low-level morphological modification and removed holes. Image intensity was rescaled (0.2–1) to optimize punctate LC3B/LAMP1 identification. Nuclei and cells were respectively discriminated using IdentifyPrimaryObjects and IdentifySecondaryObjects modules in CellProfiler. Punctate LC3B/LAMP1 with diameters of 5–75 pixels were detected using a different primary object identification module, and linked to the cells identified by the related object module. The results are shown as numbers of puncta per cell.

A pipeline was created in CellProfiler (v. 3.1.9) to quantify nuclear TFEB immunofluorescence intensity. In brief, single Z-section immunofluorescence images were converted to grayscale, then nuclei and cells were respectively identified using the IdentifyPrimaryObjects and IdentifySecondaryObjects modules. We detected TFEB dots within 1–40 pixels using the IdentifyPrimaryObjects in CellProfiler then linked them to nuclei and cells using RelateObjects. Intranuclear TFEB dots were merged using the SplitOrMergeObjects module, then the intensity of TFEB immunofluorescence emission was measured. The median integrated intensity of intranuclear TFEB is graphically represented with control cells set at 1.

### Live imaging

MIA^shNT^ and MIA^shTFEB^ cells were seeded in 96-well plates at 10,000 cells per well. The following day, lysosomes or active CTSB were respectively stained by incubating the cells for 24 h with vehicle or 10 µM gemcitabine in DMEM without phenol red. The cells were stained for 30 min with either 100 nM LysoTracker Red DND-99 (L7528; Thermo Fisher Scientific Inc.) or 1:250 Magic Red dye (#937; ImmunoChemistry Technologies LLC, Bloomington, MN, USA). Nuclei were stained with Hoechst 3342 (1 µg/mL) for 8 min. Images were acquired using ZEN Blue 2.5 software and a Zeiss Cell Discoverer 7 imaging system equipped with a Plan-Apochromat 20× and 2× Objectives Optical magnification changer. At least 30 tiles per well were captured. A pipeline was created in CellProfiler (v. 4.1.3) to quantify the LysoTracker and Magic Red stains. Briefly, the images were converted into grayscale images. Nuclei and cells were detected using the IdentifyPrimaryObjects and IdentifySecondaryObjects modules, respectively. LysoTracker/Magic Red measured as integrated intensity/cell is graphically represented with control cells set at 1.

### Cathepsin B activity

MIA^shNT^ and MIA^shTFEB^ cells (1 × 10^5^/well) were seeded in duplicate in 12-well plates for 24 h, then incubated with vehicle or 10 µM gemcitabine for 24 h. The activity of CTSB was determined using fluorometric assays (NBP2-54841; Novus Biologicals, LLC, Centennial, CO, USA) as described by the manufacturer. Briefly, the cells were disrupted in CB cell lysis buffer (50 µL/well) on ice for 10 min. Cell lysates were centrifuged for 5 min at 13,000 rpm at 4 °C, then incubated for 1 h at 37 °C with 50 µL of CB reaction buffer and 200 µM of CB substrate in black 96-well plates. Fluorescent intensity was determined using a FlexStation 3 plate reader (Molecular Devices LLC., San Jose, CA, USA) at 400 nm excitation and 505 nm emission. Relative fluorescence units (RFU) per well were normalized to the protein concentration.

### Flow cytometry

MIA^shNT^ and MIA^shTFEB^ cells (75 × 10^3^/well) were seeded in duplicate in 12-well plates overnight, then incubated with vehicle or 10 µM gemcitabine for 24 h. The cells were stained with Annexin V-PE (#CBA060; MilliporeSigma) and DAPI. Then, necrotic (Annexin V^-^/DAPI^+^), early (Annexin V^+^/DAPI^-^) and late (Annexin V^+^/DAPI^+^) apoptotic cells were discriminated using a CytoFlex 30 benchtop flow cytometer (Beckman Coulter, Inc. Brea, CA, USA). The flow cytometry data were analyzed using CytExpert software.

### Clonogenic assays

MIA^shNT^ and MIA^shTFEB^ cells (1 × 10^3^/well) were seeded in 6-well plates overnight, then incubated for 6 h with vehicle or the indicated concentrations of gemcitabine. The cells were then rinsed twice with culture media and cultured for 7–10 days. Thereafter, the cells were fixed for 10 min in 100% methanol at −20 °C, stained with 0.5% crystal violet for 1 h, and washed with PBS. Colonies in plates were visualized using the ChemiDoc Imaging System (Bio-Rad Laboratories Inc., Hercules, CA, USA) and counted using the ImageJ Colony_Area Plugin. The colony area is graphically represented with MIA^shNT^ control cells set at 1.

### Orthotopic pancreatic cancer models

Animal experiments complied with the guidelines of the Canadian Council on Animal Care, and the institutional animal care committee approved the protocols. Briefly, MIA^shNT^ or MIA^shTFEB^ cells (5 × 10^5^/20 μL of sterile 1× PBS) were orthotopically injected using a 28 gauge needle into the pancreas of 6–8 weeks old male NCG mice (Charles River Laboratories International Inc., Wilmington, MA, USA) (*N* = 7 per group). The mice were euthanized by cervical dislocation under isoflurane anesthesia 28 days later. Pancreases with embedded tumors were weighed as an indicator of tumorigenicity.

## Supplementary information

Original Data File

## Data Availability

The datasets generated during and/or analysed during the current study are available from the corresponding author on reasonable request.

## References

[1] Neoptolemos JP, Kleeff J, Michl P, Costello E, Greenhalf W, Palmer DH (2018). Therapeutic developments in pancreatic cancer: current and future perspectives. Nat Rev Gastroenterol Hepatol.

[2] Ottaiano A, Capozzi M, De Divitiis C, De Stefano A, Botti G, Avallone A (2017). Gemcitabine mono-therapy versus gemcitabine plus targeted therapy in advanced pancreatic cancer: a meta-analysis of randomized phase III trials. Acta Oncol.

[3] Rahib L, Smith BD, Aizenberg R, Rosenzweig AB, Fleshman JM, Matrisian LM (2014). Projecting cancer incidence and deaths to 2030: the unexpected burden of thyroid, liver, and pancreas cancers in the United States. Cancer Res.

[4] Ying H, Dey P, Yao W, Kimmelman AC, Draetta GF, Maitra A (2016). Genetics and biology of pancreatic ductal adenocarcinoma. Genes Dev.

[5] Pupo E, Avanzato D, Middonti E, Bussolino F, Lanzetti L (2019). KRAS-driven metabolic rewiring reveals novel actionable targets in cancer. Front Oncol.

[6] Encarnacion-Rosado J, Kimmelman AC (2021). Harnessing metabolic dependencies in pancreatic cancers. Nat Rev Gastroenterol Hepatol.

[7] Ballabio A, Bonifacino JS (2020). Lysosomes as dynamic regulators of cell and organismal homeostasis. Nat Rev Mol Cell Biol.

[8] Lawrence RE, Zoncu R (2019). The lysosome as a cellular centre for signalling, metabolism and quality control. Nat Cell Biol.

[9] Saftig P, Puertollano R (2021). How lysosomes sense, integrate, and cope with stress. Trends Biochem Sci.

[10] Boya P, Reggiori F, Codogno P (2013). Emerging regulation and functions of autophagy. Nat cell Biol.

[11] Yang S, Wang X, Contino G, Liesa M, Sahin E, Ying H (2011). Pancreatic cancers require autophagy for tumor growth. Genes Dev.

[12] Rosenfeldt MT, O’Prey J, Morton JP, Nixon C, MacKay G, Mrowinska A (2013). p53 status determines the role of autophagy in pancreatic tumour development. Nature.

[13] Yang A, Herter-Sprie G, Zhang H, Lin EY, Biancur D, Wang X (2018). Autophagy sustains pancreatic cancer growth through both cell-autonomous and nonautonomous mechanisms. Cancer Disco.

[14] Yang A, Kimmelman AC (2014). Inhibition of autophagy attenuates pancreatic cancer growth independent of TP53/TRP53 status. Autophagy.

[15] Yang A, Rajeshkumar NV, Wang X, Yabuuchi S, Alexander BM, Chu GC (2014). Autophagy is critical for pancreatic tumor growth and progression in tumors with p53 alterations. Cancer Disco.

[16] Commisso C, Davidson SM, Soydaner-Azeloglu RG, Parker SJ, Kamphorst JJ, Hackett S (2013). Macropinocytosis of protein is an amino acid supply route in Ras-transformed cells. Nature.

[17] Zhang Y, Commisso C (2019). Macropinocytosis in cancer: a complex signaling network. Trends Cancer.

[18] Geisslinger F, Muller M, Vollmar AM, Bartel K (2020). Targeting lysosomes in cancer as promising strategy to overcome chemoresistance-A mini review. Front Oncol.

[19] Fu Z, Cheng X, Kuang J, Feng H, Chen L, Liang J (2018). CQ sensitizes human pancreatic cancer cells to gemcitabine through the lysosomal apoptotic pathway via reactive oxygen species. Mol Oncol.

[20] Hashimoto D, Blauer M, Hirota M, Ikonen NH, Sand J, Laukkarinen J (2014). Autophagy is needed for the growth of pancreatic adenocarcinoma and has a cytoprotective effect against anticancer drugs. Eur J Cancer.

[21] Papademetrio DL, Cavaliere V, Simunovich T, Costantino S, Campos MD, Lombardo T (2014). Interplay between autophagy and apoptosis in pancreatic tumors in response to gemcitabine. Target Oncol.

[22] Klionsky DJ, Abdel-Aziz AK, Abdelfatah S, Abdellatif M, Abdoli A, Abel S (2021). Guidelines for the use and interpretation of assays for monitoring autophagy (4th edition)(1). Autophagy.

[23] Tan Q, Wang H, Hu Y, Hu M, Li X, Aodengqimuge (2015). Src/STAT3-dependent heme oxygenase-1 induction mediates chemoresistance of breast cancer cells to doxorubicin by promoting autophagy. Cancer Sci.

[24] Fang LM, Li B, Guan JJ, Xu HD, Shen GH, Gao QG (2017). Transcription factor EB is involved in autophagy-mediated chemoresistance to doxorubicin in human cancer cells. Acta Pharm Sin.

[25] Lamark T, Svenning S, Johansen T (2017). Regulation of selective autophagy: the p62/SQSTM1 paradigm. Essays Biochem.

[26] Jiang P, Mizushima N (2015). LC3- and p62-based biochemical methods for the analysis of autophagy progression in mammalian cells. Methods.

[27] Eliopoulos AG, Havaki S, Gorgoulis VG (2016). DNA damage response and autophagy: a meaningful partnership. Front Genet.

[28] Biancur DE, Kapner KS, Yamamoto K, Banh RS, Neggers JE, Sohn ASW (2021). Functional genomics identifies metabolic vulnerabilities in pancreatic cancer. Cell Metab.

[29] Bryant KL, Stalnecker CA, Zeitouni D, Klomp JE, Peng S, Tikunov AP (2019). Combination of ERK and autophagy inhibition as a treatment approach for pancreatic cancer. Nat Med.

[30] Kinsey CG, Camolotto SA, Boespflug AM, Guillen KP, Foth M, Truong A (2019). Protective autophagy elicited by RAF->MEK->ERK inhibition suggests a treatment strategy for RAS-driven cancers. Nat Med.

[31] Gilmartin AG, Bleam MR, Groy A, Moss KG, Minthorn EA, Kulkarni SG (2011). GSK1120212 (JTP-74057) is an inhibitor of MEK activity and activation with favorable pharmacokinetic properties for sustained in vivo pathway inhibition. Clin Cancer Res.

[32] Puertollano R, Ferguson SM, Brugarolas J, Ballabio A (2018). The complex relationship between TFEB transcription factor phosphorylation and subcellular localization. EMBO J.

[33] Marchand B, Arsenault D, Raymond-Fleury A, Boisvert FM, Boucher MJ (2015). Glycogen synthase kinase-3 (GSK3) inhibition induces prosurvival autophagic signals in human pancreatic cancer cells. J Biol Chem.

[34] He R, Wang M, Zhao C, Shen M, Yu Y, He L (2019). TFEB-driven autophagy potentiates TGF-beta induced migration in pancreatic cancer cells. J Exp Clin Cancer Res.

[35] Perera RM, Stoykova S, Nicolay BN, Ross KN, Fitamant J, Boukhali M (2015). Transcriptional control of autophagy-lysosome function drives pancreatic cancer metabolism. Nature.

[36] Zhao B, Dierichs L, Gu JN, Trajkovic-Arsic M, Axel Hilger R, Savvatakis K (2020). TFEB-mediated lysosomal biogenesis and lysosomal drug sequestration confer resistance to MEK inhibition in pancreatic cancer. Cell Death Disco.

[37] Kim JH, Lee J, Cho YR, Lee SY, Sung GJ, Shin DM (2021). TFEB supports pancreatic cancer growth through the transcriptional regulation of glutaminase. Cancers (Basel).

[38] Ropolo A, Catrinacio C, Renna FJ, Boggio V, Orquera T, Gonzalez CD (2020). A novel E2F1-EP300-VMP1 pathway mediates gemcitabine-induced autophagy in pancreatic cancer cells carrying oncogenic KRAS. Front Endocrinol (Lausanne).

[39] Guo JY, Chen HY, Mathew R, Fan J, Strohecker AM, Karsli-Uzunbas G (2011). Activated Ras requires autophagy to maintain oxidative metabolism and tumorigenesis. Genes Dev.

[40] Lock R, Roy S, Kenific CM, Su JS, Salas E, Ronen SM (2011). Autophagy facilitates glycolysis during Ras-mediated oncogenic transformation. Mol Biol Cell.

[41] Ying H, Kimmelman AC, Lyssiotis CA, Hua S, Chu GC, Fletcher-Sananikone E (2012). Oncogenic Kras maintains pancreatic tumors through regulation of anabolic glucose metabolism. Cell.

[42] Maertin S, Elperin JM, Lotshaw E, Sendler M, Speakman SD, Takakura K (2017). Roles of autophagy and metabolism in pancreatic cancer cell adaptation to environmental challenges. Am J Physiol Gastrointest Liver Physiol.

[43] Deschenes-Simard X, Gaumont-Leclerc MF, Bourdeau V, Lessard F, Moiseeva O, Forest V (2013). Tumor suppressor activity of the ERK/MAPK pathway by promoting selective protein degradation. Genes Dev.

[44] Deschenes-Simard X, Kottakis F, Meloche S, Ferbeyre G (2014). ERKs in cancer: friends or foes?. Cancer Res.

[45] Stern DF (2018). Keeping tumors out of the MAPK fitness zone. Cancer Disco.

[46] Klomp JE, Lee YS, Goodwin CM, Papke B, Klomp JA, Waters AM (2021). CHK1 protects oncogenic KRAS-expressing cells from DNA damage and is a target for pancreatic cancer treatment. Cell Rep..

[47] Hamura R, Shirai Y, Shimada Y, Saito N, Taniai T, Horiuchi T (2021). Suppression of lysosomal acid alpha-glucosidase impacts the modulation of transcription factor EB translocation in pancreatic cancer. Cancer Sci.

[48] Alderton GK (2015). Autophagy: Surviving stress in pancreatic cancer. Nat Rev Cancer.

[49] Davidson SM, Jonas O, Keibler MA, Hou HW, Luengo A, Mayers JR (2017). Direct evidence for cancer-cell-autonomous extracellular protein catabolism in pancreatic tumors. Nat Med.

[50] Gupta S, Yano J, Mercier V, Htwe HH, Shin HR, Rademaker G (2021). Lysosomal retargeting of Myoferlin mitigates membrane stress to enable pancreatic cancer growth. Nat Cell Biol.

[51] Waters AM, Der CJ (2018). KRAS: The critical driver and therapeutic target for pancreatic cancer. Cold Spring Harb Perspect Med.

[52] Brown WS, McDonald PC, Nemirovsky O, Awrey S, Chafe SC, Schaeffer DF (2020). Overcoming adaptive resistance to KRAS and MEK inhibitors by co-targeting mTORC1/2 complexes in pancreatic cancer. Cell Rep. Med.

[53] Karasic TB, O’Hara MH, Loaiza-Bonilla A, Reiss KA, Teitelbaum UR, Borazanci E (2019). Effect of gemcitabine and nab-Paclitaxel with or without hydroxychloroquine on patients with advanced pancreatic cancer: a phase 2 randomized clinical trial. JAMA Oncol.

[54] Carling PJ, Ryan BJ, McGuinness W, Kataria S, Humble SW, Milde S, et al. Multiparameter phenotypic screening for endogenous TFEB and TFE3 translocation identifies novel chemical series modulating lysosome function. Autophagy. 2022;19:692–705. 10.1080/15548627.2022.2095834 PMC9851200 35786165

[55] Vanzo R, Bartkova J, Merchut-Maya JM, Hall A, Bouchal J, Dyrskjot L (2020). Autophagy role(s) in response to oncogenes and DNA replication stress. Cell Death Differ.

[56] Bushweller JH (2019). Targeting transcription factors in cancer - from undruggable to reality. Nat Rev Cancer.

[57] Furukawa T, Duguid WP, Rosenberg L, Viallet J, Galloway DA, Tsao MS (1996). Long-term culture and immortalization of epithelial cells from normal adult human pancreatic ducts transfected by the E6E7 gene of human papilloma virus 16. Am J Pathol.

[58] Ouyang H, Mou L, Luk C, Liu N, Karaskova J, Squire J (2000). Immortal human pancreatic duct epithelial cell lines with near normal genotype and phenotype. Am J Pathol.

[59] Marchand B, Tremblay I, Cagnol S, Boucher MJ (2012). Inhibition of glycogen synthase kinase-3 activity triggers an apoptotic response in pancreatic cancer cells through JNK-dependent mechanisms. Carcinogenesis.

