# Supplementary figures and images for: Gemcitabine promotes autophagy and lysosomal function through ERK- and TFEB-dependent mechanisms
Source: Cell Death Discov. 2023 Feb 6;9:45. doi: 10.1038/s41420-023-01342-z (PMC9902516; doi:10.1038/s41420-023-01342-z)

**Supplementary Information**

**Original data files**

**Uncropped western blots**

**
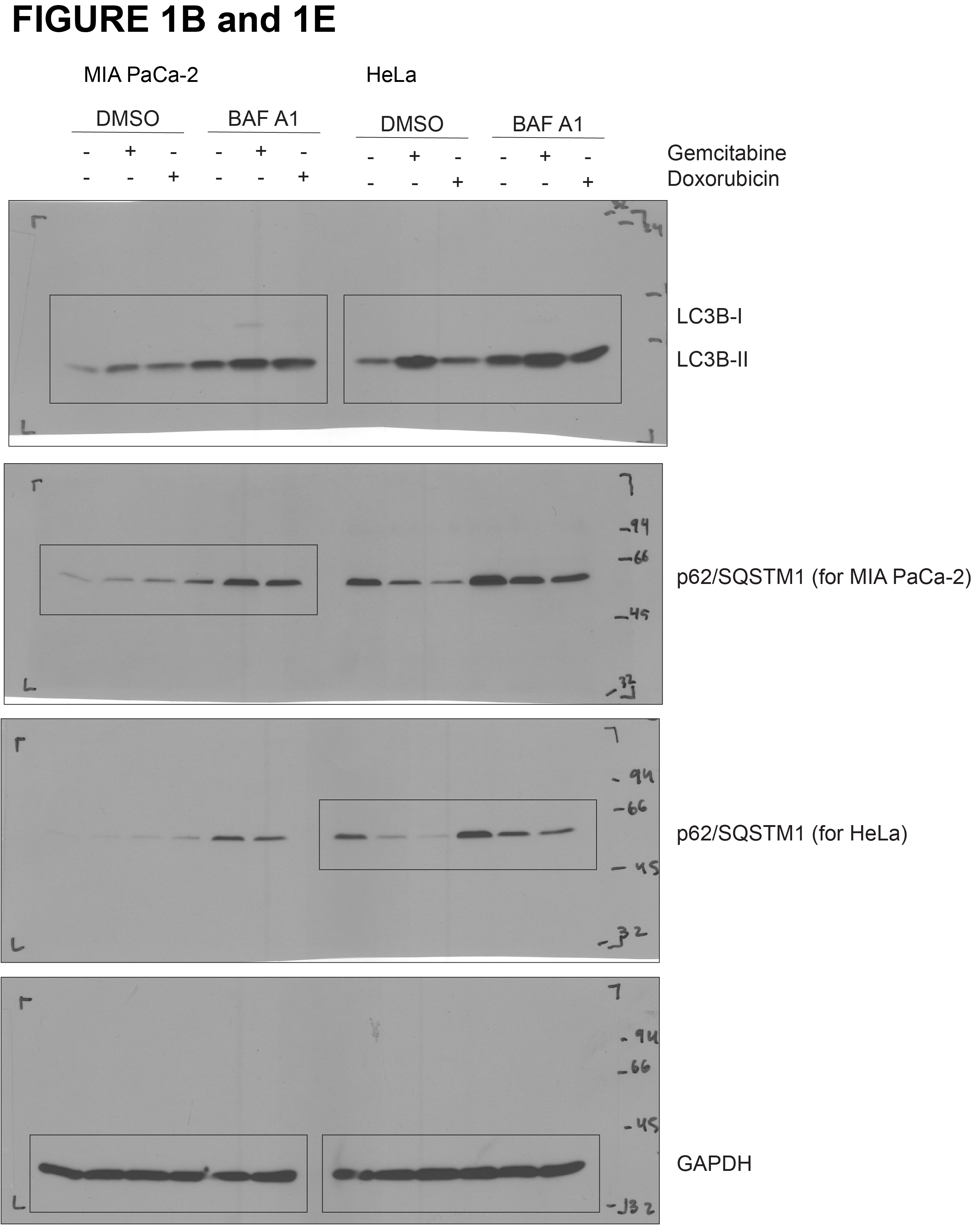
**

**
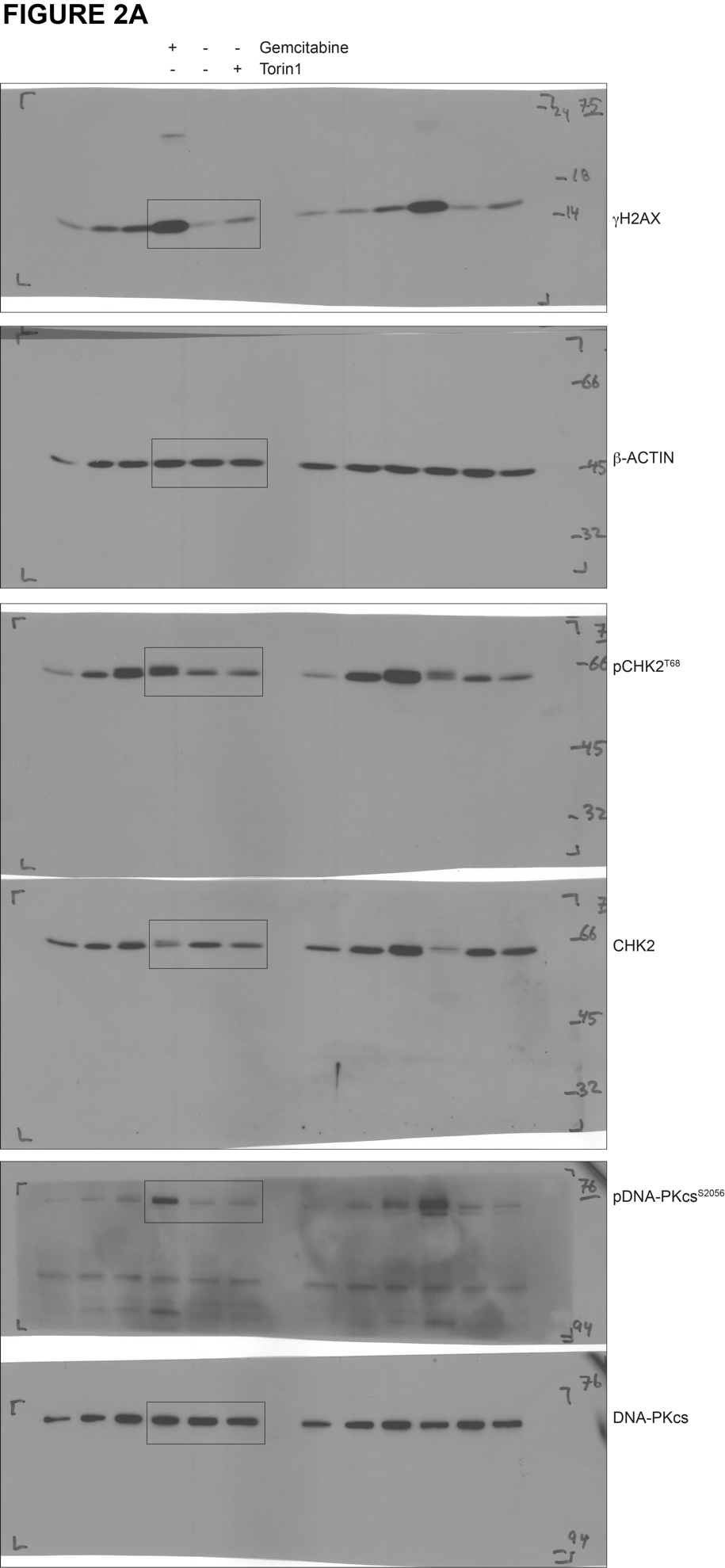
**

**
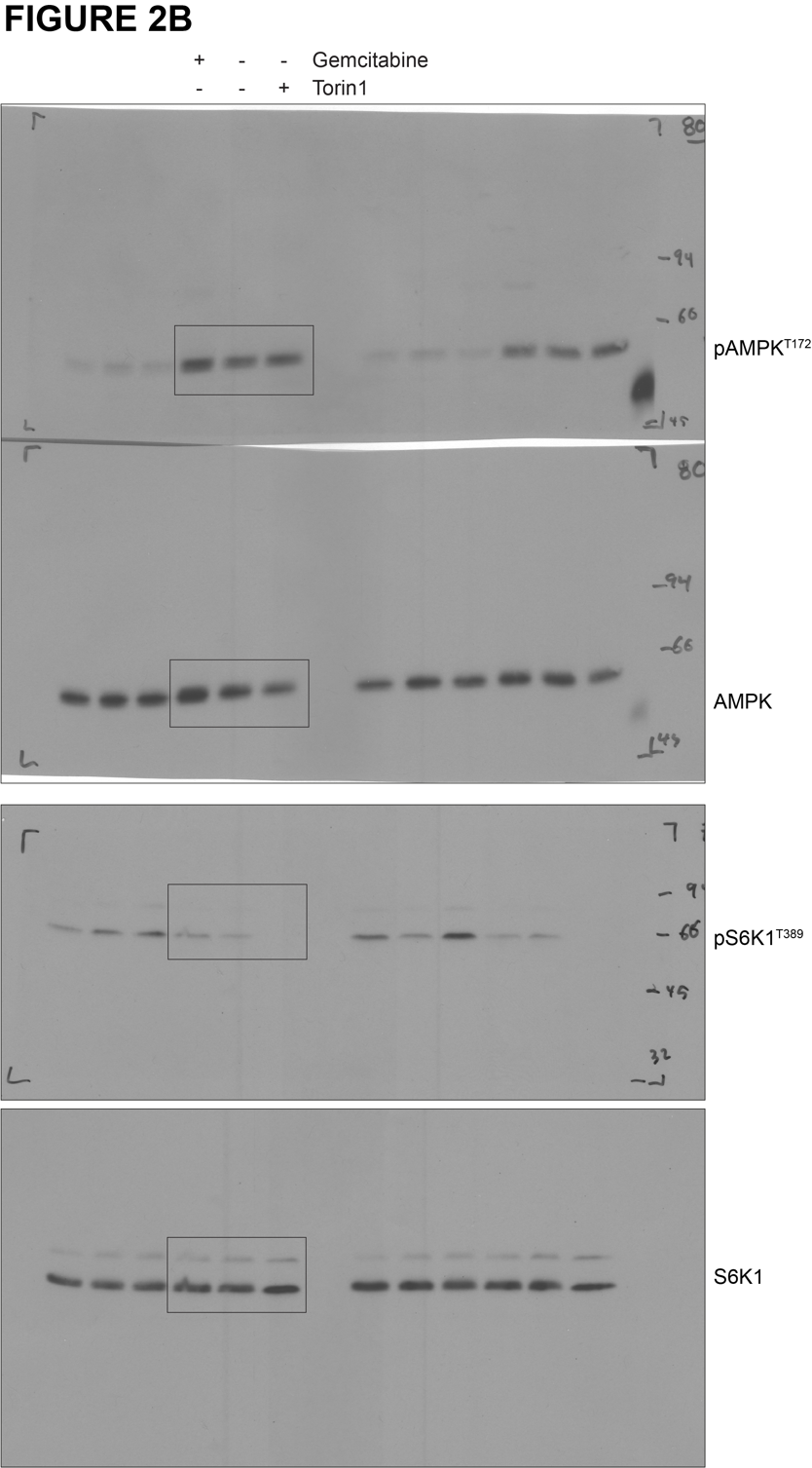
**

**
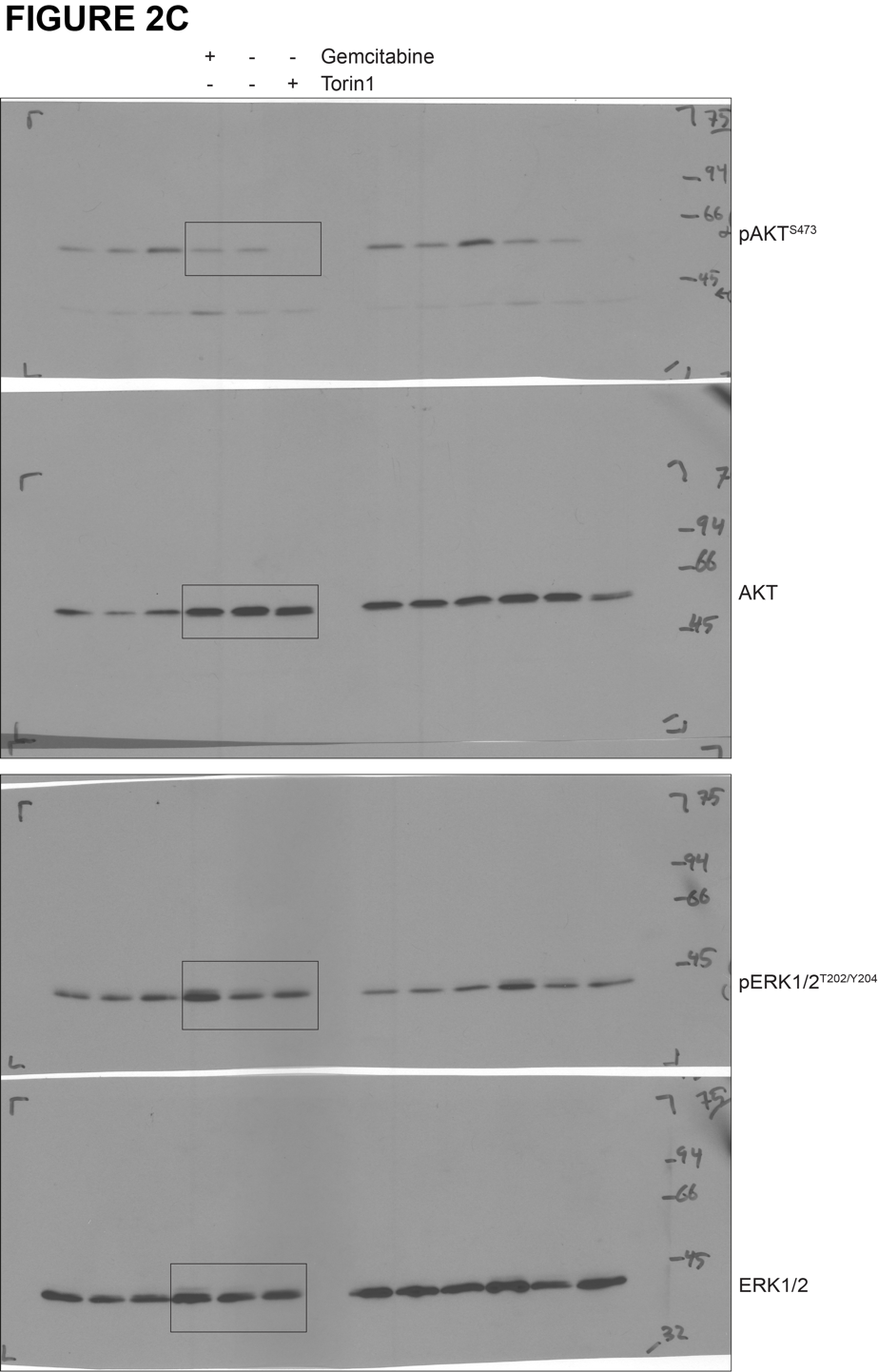
**

**
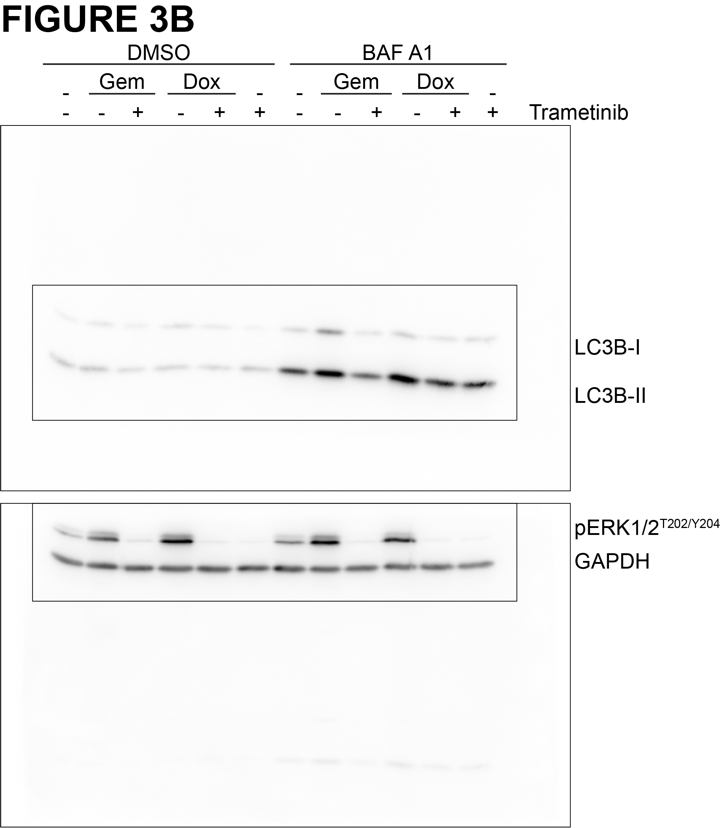
**

**
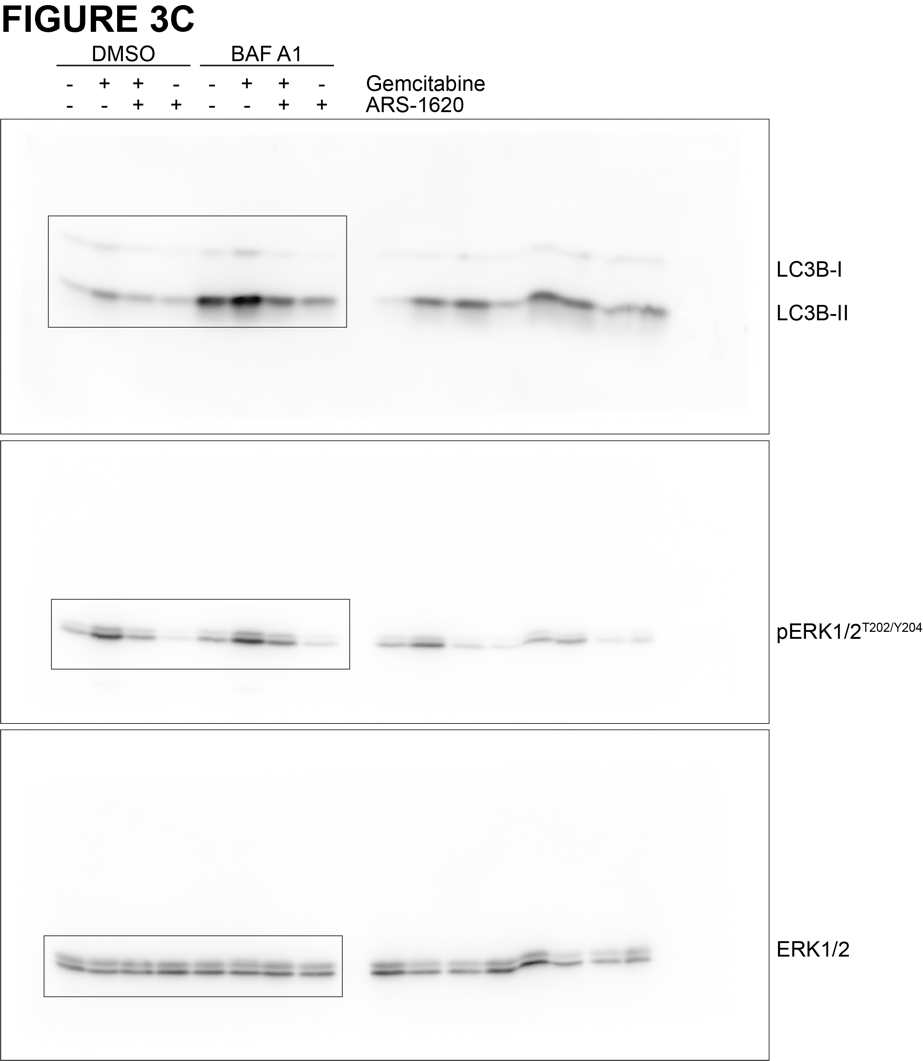
**

**
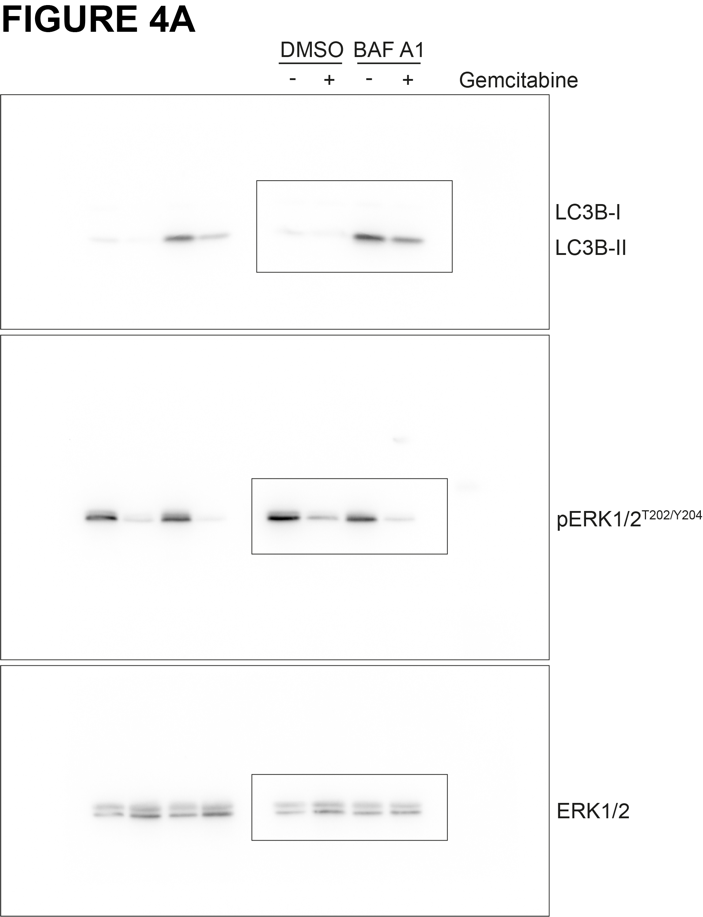
**

**
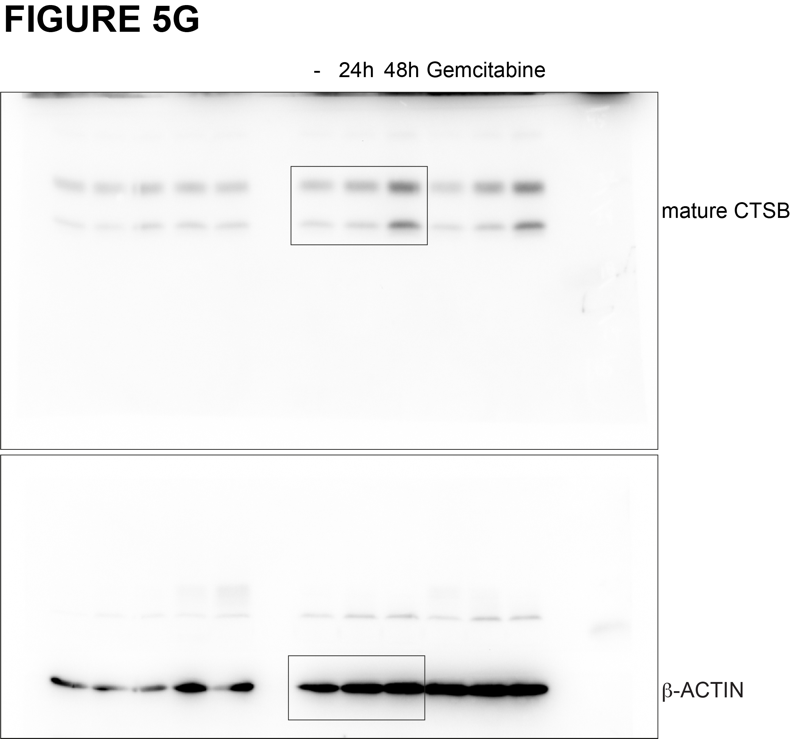
**

**
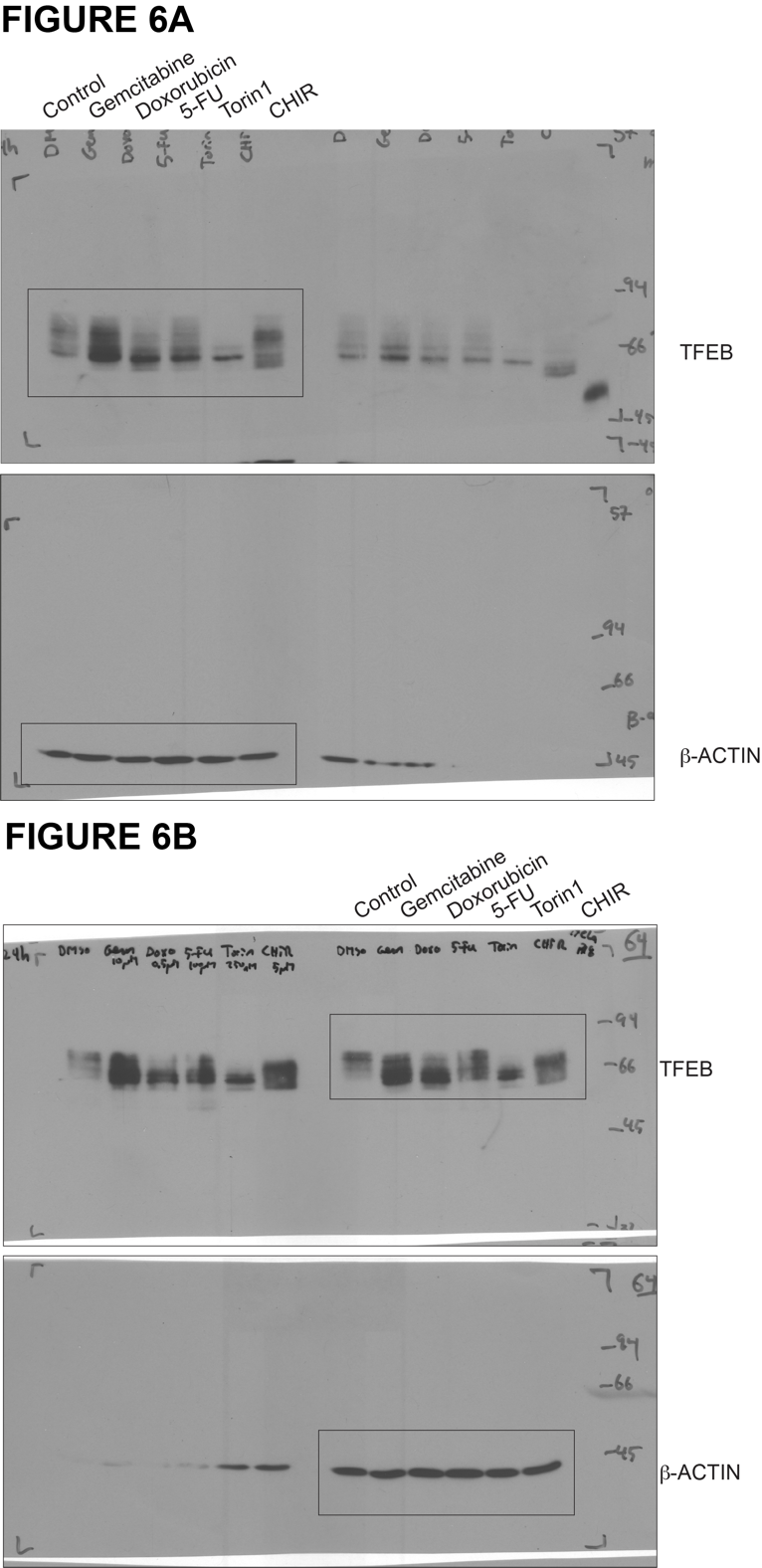
**

**
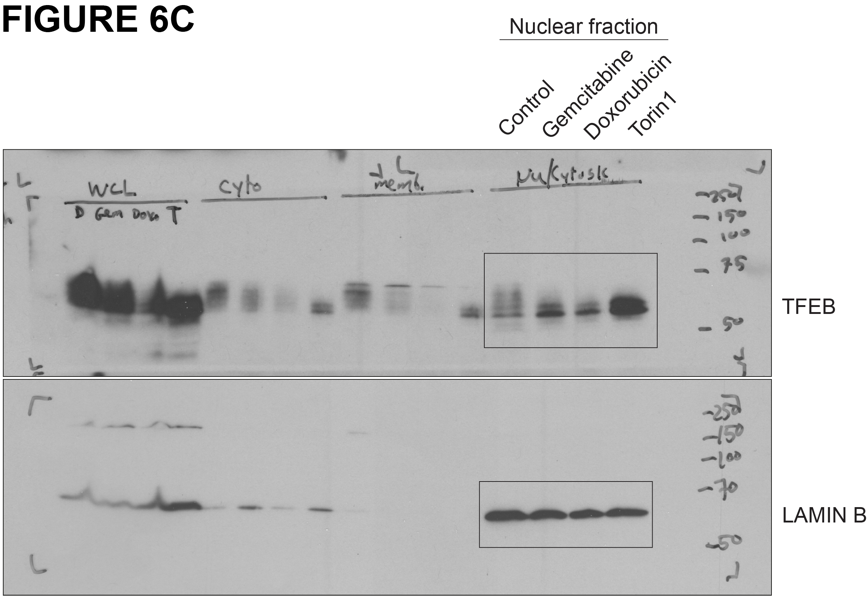
**

**
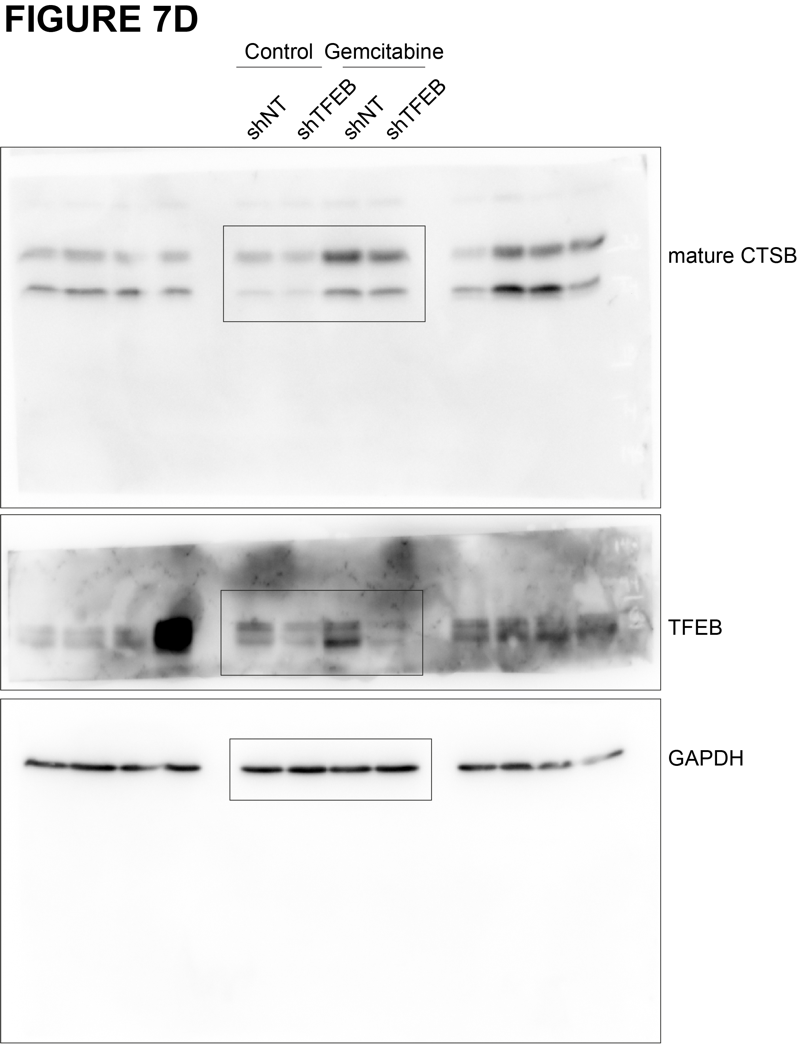
**

**
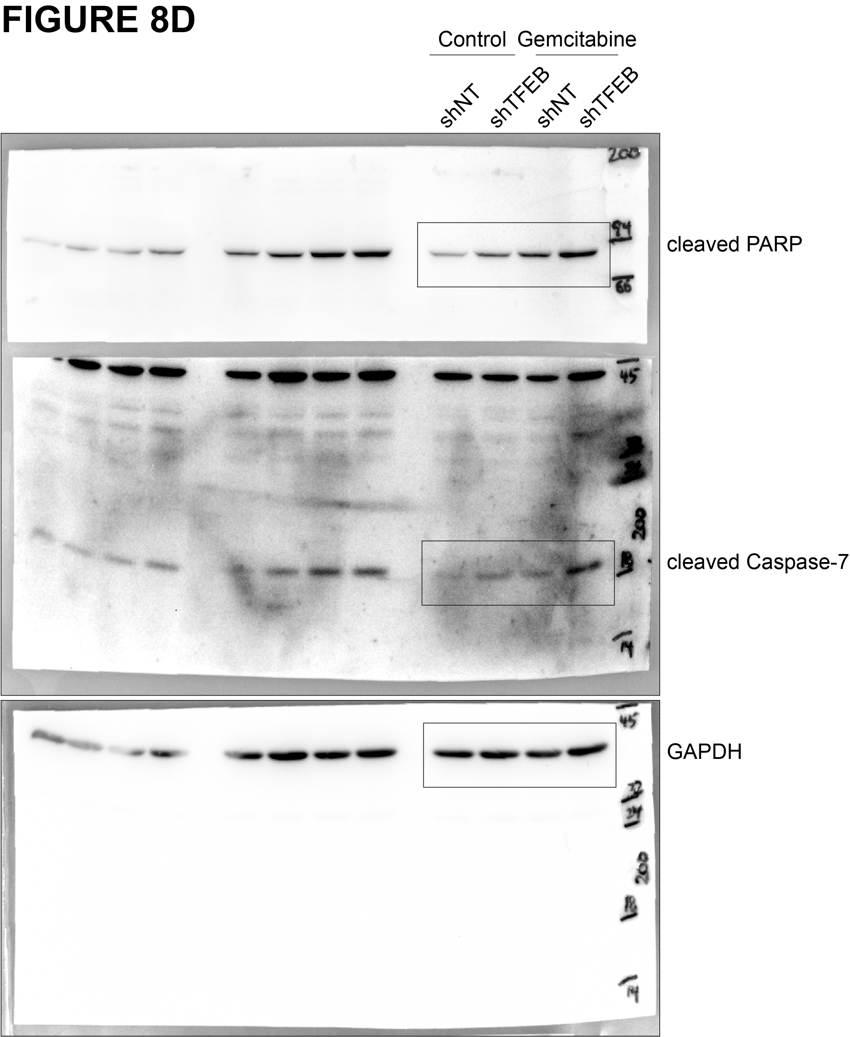
**

Supplement: Supplementary file 1 — Original Data File [file 41420_2023_1342_MOESM1_ESM.docx]
